# *Bifidobacterium* species viability in dairy-based probiotic foods: challenges and innovative approaches for accurate viability determination and monitoring of probiotic functionality

**DOI:** 10.3389/fmicb.2024.1327010

**Published:** 2024-02-02

**Authors:** Thulani Sibanda, Tlaleo Azael Marole, Ursula Louise Thomashoff, Mapitsi S. Thantsha, Elna M. Buys

**Affiliations:** ^1^Department of Consumer and Food Sciences, University of Pretoria, Pretoria, South Africa; ^2^Department of Applied Biology and Biochemistry, National University of Science and Technology, Bulawayo, Zimbabwe; ^3^Department of Biology, National of University of Lesotho, Maseru, Lesotho; ^4^Department of Biochemistry, Genetics and Microbiology, University of Pretoria, Pretoria, South Africa

**Keywords:** *Bifidobacterium*, viability, yogurt, probiotic, gut microbiota, stress response, viability improvement, next-generation methods

## Abstract

*Bifidobacterium* species are essential members of a healthy human gut microbiota. Their presence in the gut is associated with numerous health outcomes such as protection against gastrointestinal tract infections, inflammation, and metabolic diseases. Regular intake of *Bifidobacterium* in foods is a sustainable way of maintaining the health benefits associated with its use as a probiotic. Owing to their global acceptance, fermented dairy products (particularly yogurt) are considered the ideal probiotic carrier foods. As envisioned in the definition of probiotics as “live organisms,” the therapeutic functionalities of *Bifidobacterium* spp. depend on maintaining their viability in the foods up to the point of consumption. However, sustaining *Bifidobacterium* spp. viability during the manufacture and shelf-life of fermented dairy products remains challenging. Hence, this paper discusses the significance of viability as a prerequisite for *Bifidobacterium* spp. probiotic functionality. The paper focuses on the stress factors that influence *Bifidobacterium* spp. viability during the manufacture and shelf life of yogurt as an archetypical fermented dairy product that is widely accepted as a delivery vehicle for probiotics. It further expounds the *Bifidobacterium* spp. physiological and genetic stress response mechanisms as well as the methods for viability retention in yogurt, such as microencapsulation, use of oxygen scavenging lactic acid bacterial strains, and stress-protective agents. The report also explores the topic of viability determination as a critical factor in probiotic quality assurance, wherein, the limitations of culture-based enumeration methods, the challenges of species and strain resolution in the presence of lactic acid bacterial starter and probiotic species are discussed. Finally, new developments and potential applications of next-generation viability determination methods such as flow cytometry, propidium monoazide–quantitative polymerase chain reaction (PMA-qPCR), next-generation sequencing, and single-cell Raman spectroscopy (SCRS) methods are examined.

## 1 Introduction

The genus *Bifidobacterium* consists of Gram-positive bacteria belonging to the family Bifidobacteriaceae (Biavati and Mattarelli, 2015). Members of this genus are anaerobic or sometimes aerotolerant, non-spore-forming pleomorphic bacteria (Eckel et al., 2020). The taxonomy of the genus has been steadily changing over time due to advances in genomic characterization techniques, with the discovery of new species and subspecies in recent years (Lugli et al., 2018, 2021; Neuzil-Bunesova et al., 2020; Chen et al., 2021). As of the end of 2021, there were 98 documented species of the *Bifidobacterium* genus (Turroni et al., 2021). The primary ecological niche of *Bifidobacterium* species is the gastrointestinal tract (GIT) of mammals, birds, and insects (Alessandri et al., 2021). Within this diverse genus, some *Bifidobacterium* species were historically assumed to colonize only the GIT of specific animal host species (Turroni et al., 2011). However, advanced metagenomic approaches, such as bifidobacterial internally transcribed spacer (ITS) profiling, revealed that several of the *Bifidobacterium* species are ubiquitous within the GIT of different host animal species (Alessandri et al., 2021). Based on the core genome analysis, the genus is divided into 10 phylogenetic groups (*B. adolescentis*, *B. boum*, *B. pullorum*, *B. asteroides*, *B. longum*, *B. psychraerophilum*, *B. bifidum*, *B. pseudolongum*, *B. bombi* and *B. tissieri*) that partially correlate with animal host ecological niche (Alessandri et al., 2021; Duranti et al., 2021). Four of these phylogenetic groups are typical colonizers of the human GIT (Duranti et al., 2021). These include, the *B. adolescentis* group (*B. adolescentis* and *B. catenulatum* strains), the *B. longum* group (*B. breve* and *B. longum* strains), the *B. pseudolongum* group (*B. animalis* strains) and the *B. bifidum* group (*B. bifidum* strains) (Alessandri et al., 2021; Duranti et al., 2021).

Bifidobacteria are an integral component of the human gut microbiota, and their presence is associated with several health benefits (Sharma et al., 2021; Li et al., 2023). Due to their strong association with breast milk, *Bifidobacterium* species are among the earliest and most dominant colonizers of the GIT of neonates, making up to 90% of the microbiota of infants (Wong et al., 2020; Yang et al., 2021). Their relative abundance decreases in adult humans but remains stable at about 10–40% of the microbiota (Arboleya et al., 2016). In old age, the proportion decreases to about 5% (Arboleya et al., 2016). In addition to the decline in the relative abundance, there is also a change in the species diversity with age. The species, *B. breve*, *B. longum* subsp. *infantis*, and *B. bifidum* are the most dominant in infants, while *B. longum*, *B. catenulatum* and *B. adolescentis* dominate in adults (Derrien et al., 2022). Moreover, variations have also been reported among the elderly populations (Wang et al., 2015; Kato et al., 2017). For instance, the microbiota of Chinese centenarians was found to comprise of unique species, such as *B. minimum*, *B. gallinarum*/*B. pullorum*/*B. saecularmay*, and *B. mongoliense* which were absent in younger elderlies of 80–90 years (Wang et al., 2015). Due to dietary and stress factors such as antibiotic use, bifidobacterial levels in the GIT may be depleted, resulting in a dysbiosis of gut microbiota (Derrien et al., 2022). Evidence has shown that their reduction in the GIT is correlated with adverse health outcomes such as an increased risk of obesity, type 2 diabetes, allergic pathologies, irritable bowel syndrome, colorectal cancer and infections due to enteric viruses and bacterial pathogens (Xu et al., 2012; Akay et al., 2014; Taverniti and Guglielmetti, 2014; Gao et al., 2015; Kosumi et al., 2018; Wei et al., 2018; Li L. et al., 2021; Colston et al., 2022).

A sustainable approach to mitigate dysbiosis of the human gut microbiota and the attendant adverse health effects is the supplementation of bifidobacteria in foods as probiotics (He et al., 2023). According to the Food and Agriculture Organization of the United Nations [FAO], and World Health Organization [WHO] (2001) definition, probiotics are “live micro-organisms, which when consumed in adequate amounts confer a health benefit on the host.” Among the foods used as potential probiotic carriers, fermented dairy products, especially yogurt, are the most consumed. However, the sustenance of probiotic viability during processing and shelf-life of foods like yogurt is challenging. Viability is a prerequisite for probiotic functionality and therapeutic benefits (Terpou et al., 2019). The significance of viability has been demonstrated by the fact that probiotic functionalities such as antimicrobial effects, lactose intolerance relief and immune stimulation depend on cell viability (Terpou et al., 2019). Thus, for any therapeutic effects associated with probiotic intake, it is recommended that the levels of viable cells must be at least 10^6^ CFU/g of food product at the time of consumption (Nyanzi et al., 2021). Several process factors such as homogenization, mixing, heating, fermentation, and cooling in the manufacturing of fermented dairy products potentially influence *Bifidobacterium* viability.

Physicochemical stresses such as dissolved oxygen, acidic pH, homogenization pressure and storage temperature constitute the main inhibitory factors (Meybodi et al., 2020). Moreover, antagonistic effects of the starter cultures, such as the production of H_2_O_2_, could also negatively impact *Bifidobacterium* viability (Meybodi et al., 2020). Due to the poor technological robustness, many potentially beneficial *Bifidobacterium* species with superior probiotic functionalities in the human GIT cannot be effectively incorporated into fermented dairy foods. Although most of the identified *Bifidobacterium* species are from the GIT of animals, some species and strains have recently been shown to be endogenously present in fermented foods (Laureys et al., 2016; Eckel et al., 2020). So far, a few species and strains, such as the moderately aerotolerant *B. animalis* subsp. *lactis* are used in the commercial production of probiotic foods (He et al., 2023). The non-aerotolerant strains cannot be used due to their susceptibility to oxygen exposure.

Accurate enumeration of viable *Bifidobacterium* spp. in dairy products is a critical factor in probiotic quality assurance. Specific enumeration of *Bifidobacterium* viability in yogurt is complicated by the co-occurrence of lactic acid bacteria (LAB) starter cultures. In mixed species products with *Lacticaseibacillus rhamnosus* and *Lactobacillus acidophilus* strains, the widely used medium for enumeration of *Bifidobacterium* spp. [De Man, Rogosa and Sharpe (MRS) agar supplemented with neomycin, nalidixic acid, lithium chloride and paromomycin (MRS-NNLP agar)] could not select for *Bifidobacterium* spp. (Van de Casteele et al., 2006; Ashraf and Smith, 2015). Moreover, probiotic functionality is strain and species-specific, yet culture-based methods are unable to selectively differentiate between individual species and strains of bifidobacteria (Hagen and Skelley, 2019; Yoon et al., 2021). This paper reviews the subject of *Bifidobacterium* spp. viability and its significance in probiotic functionality. Given that yogurt is a widely consumed dairy product that is globally accepted as a delivery vehicle for probiotics, the paper focuses on the factors that influence *Bifidobacterium* spp. viability during the manufacturing as well as the strategies for viability retention. Furthermore, the article explores the next-generation methods for *Bifidobacterium* spp. viability determination and their applications in industrial probiotic viability quality assurance. The review was primarily based on literature published in the past 15 years, However, some earlier key studies with an enduring relevance and impact on the subject were selectively incorporated.

## 2 Probiotic functionality of *Bifidobacterium* species

A significant quantity of *in vitro* and *in vivo* evidence has demonstrated the probiotic functionalities of *Bifidobacterium* spp. (Konieczna I. et al., 2012; Groeger et al., 2013; Turroni et al., 2014; Din et al., 2020; Shang et al., 2020; Zhang et al., 2020; van der Hee and Wells, 2021; Schiweck et al., 2022; Álvarez-Mercado et al., 2022; He et al., 2023). These diverse probiotic functionalities include the enhancement of the host immune system, protection against communicable and non-communicable diseases, as well as improvement of nutritional metabolism (He et al., 2023). The immunomodulatory properties of *Bifidobacterium* species include the stimulation of both innate and adaptive immune defense systems (He et al., 2023). Some of the compelling empirical evidence for the immunostimulatory effects was demonstrated in immunosuppressed mice gavaged with *B. bifidum* strains, in which the oral administration of the probiotic resulted in increased secretion of immunoglobulin A and enhanced production and activity of lymphocytes, natural killer cells and macrophages (Turroni et al., 2014; Shang et al., 2020). In addition, experimental evidence in mice models, epithelial cell lines and human trial studies has shown that species such as *B. longum* subsp. *infantis*, *B. animalis* subsp. *lactis* and *B. infantis* can offer protection against chronic gastrointestinal inflammatory diseases such as inflammatory bowel disease and other non-enteric inflammatory diseases like autoimmune hepatitis (Konieczna I. et al., 2012; Groeger et al., 2013; Din et al., 2020; Zhang et al., 2020; Álvarez-Mercado et al., 2022). The protective mechanism is attributed to the ability of *Bifidobacterium* species to inhibit the release of proinflammatory cytokines while stimulating the release of anti-inflammatory cytokines (Konieczna I. et al., 2012; Zhang et al., 2020; Álvarez-Mercado et al., 2022; He et al., 2023). A further beneficial function of *Bifidobacterium* species relates to their role in the metabolism of dietary and human-derived heteroglycans (Li et al., 2023). Dietary heteroglycans such as arabinoxylans, pectin, and inulin are plant-derived components of dietary fiber that are not metabolized by human digestive enzymes (Kelly et al., 2021). Human-derived heteroglycans include mucin and human milk oligosaccharides (HMOs) (Luo et al., 2021). Except for a few other genera, *Bifidobacterium* species are the most prominent part of the gut microbiota capable of metabolizing heteroglycans (Li et al., 2023). The fermentation of heteroglycans has profound implications for health-promoting functions (Li et al., 2023). Among the fermentation products, short-chain fatty acids (SCFAs) such as acetate, propionate, butyrate and valerate that are linked to numerous beneficial effects (Parada Venegas et al., 2019; Schiweck et al., 2022). Using a murine model, Zhang et al. (2020) showed that oral intake of *B. animalis* subsp. *lactis* increased the concentration of fecal butyric acid in mice with experimentally induced autoimmune hepatitis. An abundance of evidence has shown that SCFAs are central to the regulation and induction of the immune system (van der Hee and Wells, 2021; Schiweck et al., 2022). They function as signaling molecules through cell surface G-protein coupled receptors (GPCRs) to control immune and metabolic functions (van der Hee and Wells, 2021; Li et al., 2023).

## 3 Viability as a necessity for probiotic functionality

As enunciated in the original definition, viability is a primary criterion for describing an organism as a probiotic (Food and Agriculture Organization of the United Nations [FAO], and World Health Organization [WHO], 2002). However, there have been many scientific reports and reviews indicating that many of the known health benefits previously ascribed to live probiotics can also be exhibited by their metabolites and/or non-viable cells (Geraldo et al., 2020; Vallejo-Cordoba et al., 2020; Martorell et al., 2021). Previous experiments comparing the physiological functionalities of live and heat-killed *B. breve* in murine models concluded that while both heat-killed and live cells exhibited similar activities in the suppression of pro-inflammatory cytokine secretion, live cells had a more significant effect on the regulation of intestinal metabolism (Sugahara et al., 2017). Similar comparisons of protective properties of heat-inactivated and live *B. longum* subsp. longum and *B. animalis* subsp. *lactis* on ovalbumin-sensitized mice and cultured Caco-2 cells, respectively, showed that live cells exhibited a stronger inflammation-suppressing effect and increased barrier-integrity of epithelial cells (Castro-Herrera et al., 2020; Pyclik et al., 2021). Despite this recent evidence, it is undeniable that the requirement for viability continues to be the standard for the incorporation of probiotics into functional foods. While some physiological effects of probiotics, such as immunomodulatory properties, can be elicited by bacterial cell components such as lipoteichoic acids and peptidoglycan, some functions are dependent on metabolic activity and thus are a product of viable cells (Castro-Herrera et al., 2020). Several aspects of *Bifidobacterium* spp. probiotic functionality depend on their metabolic activities and secretion of enzymes and bioactive metabolites. A quintessential illustration of the viability-dependent function of *Bifidobacterium* spp. in gut health is their fermentation of dietary heteroglycans and human-derived oligosaccharides (Luo et al., 2021; Li et al., 2023). Apart from the direct benefits of such metabolism of complex carbohydrates, the unique carbohydrate-active enzymes of bifidobacteria enable them to support the growth and survival of other members of the gut microbiota through cross-feeding (An et al., 2014). *In vitro* experiments with co-cultures of *Bifidobacterium* and other genera of the human gut microbiota, such as *Faecalibacterium*, *Eubacterium* and *Anaerostipes* on fructooligosaccharide substrates, showed an enhanced production of butyrate, a bioactive SCFA (Belenguer et al., 2006; Rios-Covian et al., 2015; Kim H. et al., 2020). Thus, through synergistic metabolic interactions with other members of the gut microbiota, viable *Bifidobacterium* spp. can result in the amplification of biological signals that lead to enhanced probiotic functionality.

### 3.1 Stress factors affecting viability during yogurt processing and shelf-life

**FIGURE 1:**
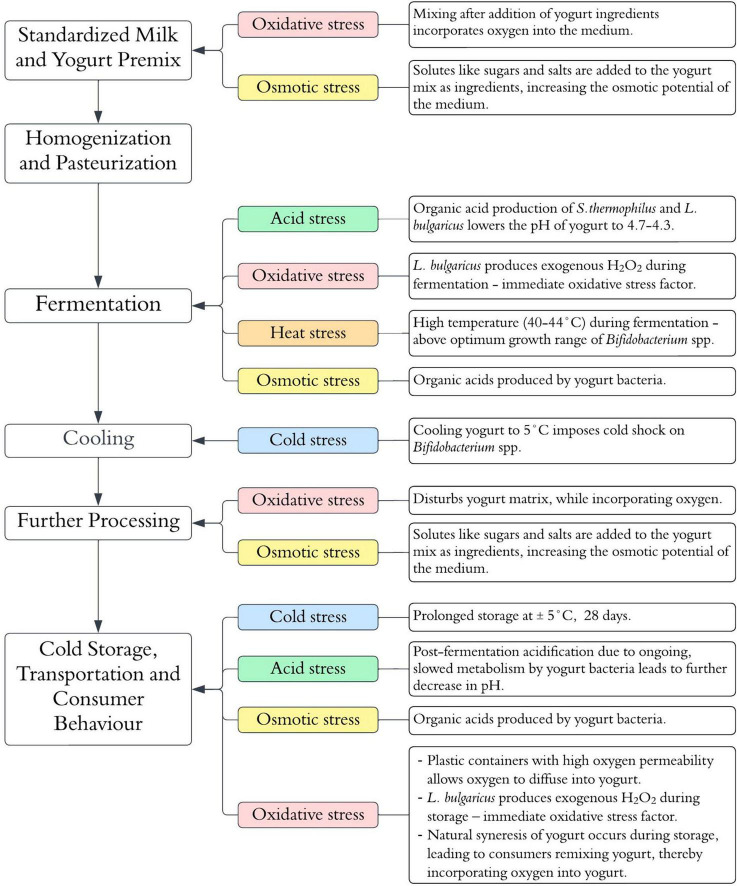
Stress factors potentially influencing *Bifidobacterium* spp. viability during yogurt manufacturing and shelf-life.

**FIGURE 2:**
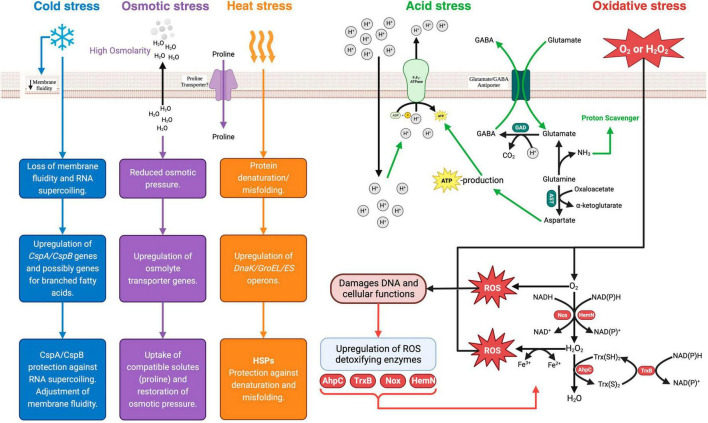
*Bifidobacterium* spp. stress response mechanisms. Csp, cold shock protein; Hsp, heat shock protein; GAD, glutamate decarboxylase; AST, aspartate transaminase; GABA, γ-amino butyric acid; EPS, exopolysaccharide; Nox, NADH oxidase; HemN, oxygen-independent coproporphyrinogen III oxidase; AhpC, Alkyl hydroperoxide reductase C-subunit; Trx(S)_2_, oxidized thioredoxin; Trx(SH)_2_, reduced thioredoxin; TrxB, thioreductase-like protein. Created with BioRender.com.

Fermented dairy products such as yogurts, cheeses, acidified milks and kefir are the most known category of food-based probiotic carrier systems for the human intake of *Bifidobacterium spp*. (Terpou et al., 2019; González-Orozco et al., 2022). Above all, the ability to survive the fermentation and associated processes during the production of these foods ultimately determines the number of viable *Bifidobacterium* spp. that reach the GIT, and the therapeutic benefit derived therefrom (Meybodi et al., 2020). This paper focuses on the viability of *Bifidobacterium* spp. in yogurt as an archetypal dairy-based probiotic carrier. Several physical and chemical stress factors associated with the yogurt manufacturing process and the subsequent storage period of the shelf-life impose adverse effects on *Bifidobacterium* viability (Meybodi et al., 2020). The sources of the physical and chemical stress factors associated with the yogurt manufacturing process are summarized in Figure 1. The ensuing subsections of the paper explore the physiological and genetic responses of *Bifidobacterium* spp. to the main stress factors associated with yogurt production (acid, osmotic, heat, oxidative and cold stress). A summary of the elucidated and postulated mechanisms is illustrated in Figure 2.

#### 3.1.1 Acid stress

In most cases, probiotic *Bifidobacterium* spp. are incorporated into yogurt together with the starter culture at the onset of fermentation. As a neutrophile, the typical optimum pH for *Bifidobacterium* spp. growth is 6.5–7.0 (Biavati and Mattarelli, 2015). Given the low pH conditions in yogurt, the survival of *Bifidobacterium* spp. depend on the ability to activate an acid tolerance response needed to maintain intracellular pH homoeostasis. The mechanisms of acid stress response have been well elucidated and involve the increased expression of the proton-translocating F_1_F_0_-ATPase (Sánchez et al., 2007; Waddington et al., 2010; Jin et al., 2012). The F_1_F_0_-ATPase is an active proton pump whose activity results in the exclusion of protons using energy derived from the hydrolysis of ATP (Fiocco et al., 2020). In addition to the F_1_F_0_-ATPase-dependent acid tolerance response, other mechanisms include the alkalization of the cytoplasm through processes that consume intracellular H^+^ protons (Fiocco et al., 2020). These include the glutamate decarboxylase (GAD) and branched-chain amino acid metabolism pathways, that generate ammonia (Schöpping et al., 2022a). The GAD system comprises of the enzyme glutamate decarboxylase (GadB) and an antiporter (GadC) encoded by the *GadB* and *GadC* loci, respectively (Yunes et al., 2016). In this pathway, glutamate is converted by the activity of GadB to γ-aminobutyrate (GABA), with the consumption of H^+^ protons and the release of CO_2_ (Duranti et al., 2020). Ammonia production is thought to play a crucial role in maintaining intracellular pH equilibrium by functioning as a proton scavenger (Wei et al., 2019). When exposed to acidity, acid-sensitive strains such as *B. longum* exhibit increased production of enzymes responsible for branched-chain amino acid (BCAA) biosynthesis (Wei et al., 2019). Furthermore, sulfur-containing amino acids, such as cysteine and methionine, are hypothesized to play a role in the acid stress response of *Bifidobacterium* spp. (Sánchez et al., 2007; Schöpping et al., 2022b). An analysis of the proteome of low pH adapted mutants of *B. longum* identified a higher constitutive presence of methionine synthase, cystathionine gamma-lyase and cystathionine gamma-synthase compared to the unadapted wild type (Sánchez et al., 2007). All these enzymes are involved in additional pathways of NH_3_ generation and alkalization of the cytoplasm (Schöpping et al., 2022b). Furthermore, the acid tolerance response in *B. longum* strains has also been linked to an overall increase in cell envelope components under sub-lethal and lethal acid environments (Jin et al., 2015). Higher transcription rates were reported for genes associated with the synthesis of peptidoglycan, exopolysaccharides, and undecaprenyl-PP (UND-PP) in *B. breve* adapted at pH 3.2 (Jin et al., 2015). The high synthesis of peptidoglycan under acid stress conditions is postulated to strengthen the cell wall structure and provide protection against acid stress-induced cell damage (Jin et al., 2015). Moreover, acid stress induces changes in the fatty acid profiles of the cell membrane with a shift toward long-chain fatty acids (C16:0 and C18:0) (Wei et al., 2019).

#### 3.1.2 Oxidative stress

Processes that allow oxygen diffusion into the milk or yogurt during manufacturing include stirring, homogenization, mixing and agitation (Figure 1). In addition, the packaging of yogurt in oxygen-permeable plastic containers can lead to an increased dissolved oxygen content over the storage period of the shelf life (Cruz et al., 2013). As anaerobes, *Bifidobacterium* spp. are intrinsically sensitive to oxygen and its derived reactive oxygen species (ROS). However, the sensitivities differ within the genus. Species such as *B. animalis* subspp. *lactis*, *B. asteroides*, *B. minimum* and *B. indicum*, are considered aerotolerant, while species such as *B. boum* and *B. thermophilum* are considered hyper-aerotolerant (Kawasaki et al., 2018). The primary response mechanisms to oxidative stress are based on the production of enzymes that can detoxify ROS. Except for a few aerotolerant species like *B. asteroides* and *B. indicum*, *Bifidobacterium* spp. generally lack the genes for the primary antioxidant enzymes like catalase and superoxide dismutase (Zuo et al., 2018). However, some *Bifidobacterium* spp. contain some inducible enzymes that can detoxify ROS (Schöpping et al., 2022b). One such enzyme is alkyl hydroperoxide reductase catalytic subunit C (AhpC) (Zuo et al., 2014). AhpC is a peroxidase component of alkyl hydroperoxide reductase enzymes systems that are found in many prokaryotes (Zuo et al., 2014). It functions together with the flavoprotein disulfide reductase (AhpF) to covert H_2_O_2_ to alcohol and water (Zuo et al., 2014). In some facultative anaerobes such as *E. coli*, AhpC is the main ROS detoxifying enzyme instead of catalase (Ma and Payne, 2012). In support of the hypothesis of inducible oxidative response systems in bifidobacteria, Xiao et al. (2011), observed that in aerobically grown *B. longum*, AhpC was one of the upregulated proteins together with DNA oxidative damage-protective proteins such as pyridine nucleotide-disulfide reductase (PNDR) and ribonucleotide reductase (NrdA). Similarly, Satoh et al. (2019), identified a thioredoxin reductase (TrxR) whose expression was induced by oxygen exposure in *B. bifidum*. Subsequently, TrxR was identified to be a reductase homologue of AhpF that interacted with AhpC *in vitro* to achieve H_2_O_2_ reduction. Hence, in *B. bifidum*, the oxidative stress response possibly involves an increased expression of thioredoxin reductase and the AhpF-AhpC H_2_O_2_ degradation system (Satoh et al., 2019).

#### 3.1.3 Osmotic stress

To date, only a few studies have tried to elucidate the bifidobacterial osmoregulatory system (Cui et al., 2022; Zhang et al., 2022). Cui et al. (2016) experimentally determined the osmolarity limits for the growth of *Bifidobacterium spp*. to range from 850 to 1,300 mosM kg^–1^. The presence of solutes in yogurt (Figure 1) results in a hyperosmotic extracellular environment that can lead to the loss of water from bacterial cells, and consequently a loss of turgor pressure and plasmolysis (Brauer et al., 2023). Based on the established osmoregulatory systems in other probiotic bacterial genera that involve the intracellular accumulation of compatible solutes such as trehalose, glycine betaine and amino acids such as proline, Zhang et al. (2022) and Cui et al. (2022) sought to characterize the osmoregulatory mechanisms in *B. bifidum* and *B. longum*, respectively. Using a genome and metabolome analysis, hyper-osmotolerant mutants of *B. bifidum* were observed to have an increased accumulation of amino acids, especially proline, compared to non-osmotically adapted wild types (Zhang et al., 2022). As no potential proline transporter proteins were identified in the organism, the proposed osmotic protection mechanism was that proline accumulation occurred through an endogenous synthesis from glutamic acid (Zhang et al., 2022). In contrast, Cui et al. (2022) observed that when the osmotic pressure of a culture environment was increased, the addition of proline substantially improved the survival of *B. longum*, suggesting the presence of proline transporter proteins in this organism.

#### 3.1.4 Heat stress

Except for a few thermophilic species like *B. thermacidophilum*, bifidobacteria are generally mesophilic organisms with an optimum growth temperature range of 37–41°C (Biavati and Mattarelli, 2015). Hence, a yogurt fermentation, which typically occurs at 40–44°C, can impose a mild heat stress on probiotic *Bifidobacterium* species. In general, the physiological effects of mild heat challenges on bacterial cells include the destabilization of non-covalent molecular bonds, ribosome dysfunctionality, and protein denaturation (Fiocco et al., 2020). Notably, apart from the earlier work by Ventura et al. (2004)^2005^ and Rezzonico et al. (2007), there has not been any studies on the bifidobacterial heat stress response systems in recent years. Like other prokaryotes, the heat stress response of *Bifidobacterium* spp. involves the increased production of heat shock proteins (HSPs) (Ventura et al., 2011). HSPs are ubiquitous and conserved proteins across the prokaryotic kingdom. They are encoded in two operons (*dnaK* and *groEL-groES*) and function as chaperones that protect physiological proteins against misfolding under conditions of heat stress (Hu et al., 2022). A transcriptomic analysis of *B. longum* exposed to heat shock treatment at 50°C found that the *dnaK* operon-encoded molecular chaperones (DnaK, GrpE, DnaJ) were the main HSPs produced (Rezzonico et al., 2007). The second class of molecular chaperones (GroEL and GroES) encoded by the *groEL-groES* operon were less expressed in *B. longum* in response to mild heat stress (Rezzonico et al., 2007). Studies on *B. breve* heat stress response showed that the GroEL and GroES chaperones are required for mild heat shock survival while dnaK, GrpE, DnaJ chaperones are necessary for survival under extreme heat stress (Ventura et al., 2004, 2005).

#### 3.1.5 Cold stress

At the end of fermentation, yogurt is typically cooled to approximately 5°C, to allow for gel setting and inhibit the growth and metabolic activities of the yogurt bacteria (Sfakianakis and Tzia, 2014). In addition to a drastic drop in temperature after fermentation, the yogurt is further stored at cold temperatures for the duration of the shelf-life, typically 28 days. Except for a few species, such as *B. mongoliense* and *B. psychraerophilum*, which can grow under cold conditions, most *Bifidobacterium* spp., minimum growth temperature is 25–28°C (Biavati and Mattarelli, 2015). The physiological effects of cold stress in mesophilic bacteria include the changes in the cell membrane from an elastic liquid crystalline state to a rigid gel-phase state that impairs nutrient uptake, and the stabilization of nucleic acid secondary structures that impede DNA replication and protein synthesis (Phadtare, 2004). So far, the molecular mechanisms behind the cold stress response of *Bifidobacterium* spp. have not specifically been elucidated. However, based on knowledge from other Gram-positive organisms, the adjustment of membrane fluidity through the incorporation of unsaturated anteiso-branched-chain fatty acids (BCFA) is one of the mechanisms of bacterial cold stress adaptation (Yoon et al., 2015). A second mechanism of bacterial cold stress adaptation involves the increased production of cold shock proteins (CSPs) that act as RNA chaperones to prevent supercoiling and facilitate effective translation under cold conditions (Phadtare, 2004). CPSs belong to a family of small, highly conserved, structurally related proteins widely distributed in the prokaryotic kingdom (Phadtare, 2004). So far, a few studies have observed the presence of CSP gene homologs (*CspA* and *CspB*) in bifidobacterial genomes (Ventura et al., 2004; Rezzonico et al., 2007; Schöpping et al., 2022b). Interestingly, *CspA* expression in *B. breve* was activated by heat stress exposure together with the *groEL-groES* operon, suggesting a heat-induced co-transcription of both genes (Ventura et al., 2004).

## 4 Strategies for *Bifidobacterium* viability retention and enhancement in dairy probiotic foods

### 4.1 Process modification

The dissolved oxygen content of milk is a crucial factor influencing *Bifidobacterium* viability in yogurt. Hence, processes targeted at reducing the dissolved oxygen content of milk before fermentation may present some rational chances of viability retention. A comparative summary of these methods is given in Table 1. Previous studies have investigated the use of gasses like nitrogen to achieve milk deaeration (Bolduc et al., 2006; Ebel et al., 2011). The bubbling of pasteurized milk with a gas mixture of N_2_ and 4% (v/v) H_2_ (N_2_-H_2_) for 4 h at a flow rate of 20 mL/min decreased the redox potential of milk from +440 mV to +350 mV and −300 mV, respectively (Ebel et al., 2011). When the de-aerated milk was fermented by yogurt starter cultures together with *B. bifidum*, the fermented products made from milk treated with N_2_H_2_ had higher survival of *B. bifidum* during storage, and the treatment had no adverse effects on the fermentation kinetics and starter cultures (Ebel et al., 2011). In a similar deaeration treatment with N_2_, the dissolved oxygen concentration of milk was reduced from an average of 6.7 ppm to 0.3 ppm (Bolduc et al., 2006).

**TABLE 1:** Methods for reducing the redox potential of milk and yogurt for the enhancement of *Bifidobacterium* spp. viability.

Category	Method	Description	Strengths and limitations	References
Process modification	Deaeration	Use of N_2_ and H_2_ gas to purge O_2_ from pasteurized milk for yogurt making	Effective at reducing the redox potential of milk (as low as −300 mV). No negative effects on fermentation kinetics. No effect on milk odor, color and taste.	Ebel et al., 2011; Roussel et al., 2022
	Electroreduction	Lowering of milk redox potential through electrolysis	Can lower milk redox potential to −300 mV. Redox potential is unstable. The low redox potential can only last up 7 days	Bazinet et al., 2009; Roussel et al., 2022
Reducing agents	L-Cysteine	Reducing agent	Strong reducing agent capable of maintaining a negative redox potential in yogurt for 30 days Sulfur taste effect limits use in yogurt	Dave and Shah, 1997a; Meybodi et al., 2020
	Ascorbic acid	Reducing agent	Instability limits the antioxidant potential	Dave and Shah, 1997b
	Oxygen-scavenging *Lactococcus lactis* strains	*Lac. lactis* has a strong reducing ability through cell surface thiol groups and membrane NADH dehydrogenases.	*Lac. lactis* is a dairy starter culture and probiotic Potential for enhanced probiotic benefit as it can complement *Bifidobacterium* spp.	Tachon et al., 2009; Michelon et al., 2010; Ayivi and Ibrahim, 2022
Redox enzymes	Glucose oxidase/Catalase system	Glucose oxidase achieves the removal of O_2_ from milk through oxidation of glucose with the resultant H_2_O_2_ removed by catalase	Glucose oxidase/Catalase system is the compatible with many food applications	Cruz et al., 2012a; Dubey et al., 2017

An alternative method of lowering redox potential is electroreduction. This is a physical treatment involving voltage application to reduce the redox potential by electrolysis (Roussel et al., 2022). It is an efficient process of decreasing the redox potential of milk. Through the application of a voltage of −1.55 V through a milk sample for 40 minutes, Bolduc et al. (2006) reported a reduction in redox potential of milk from > + 200 mV to < −300 mV and a decrease in dissolved oxygen concentrations to between 2–3 ppm from 6.7 ppm in untreated milk. When treated by electroreduction, it is possible to maintain a negative redox potential in milk for up to 7 days (Bazinet et al., 2009).

Another technological process modification considered a potential method for viability improvement is high-pressure homogenization (HPH). HPH is a non-thermal milk preservation method in which the milk is exposed to pressure above 100 MPa (Massoud et al., 2016). Experiments with *B. lactis* have shown that an increase in homogenization pressure from 100 to 200 MPa, combined with increasing temperature from 50 to 70°C, led to a significant improvement in the viability of the probiotic in the resultant yogurt (Massoud et al., 2015). The improvement in viability has been attributed to the increase in free amino acids needed for probiotic nutrition (Massoud et al., 2016). Most significantly, the release of cysteine has a positive effect on *Bifidobacterium* viability.

An additional approach to reducing the redox potential of milk and yogurt is using *Lactococcus lactis* as an oxygen scavenger (Tachon et al., 2009). *Lac. lactis* is known for its strong ability to decrease the redox potential of milk to as low as −220 mV (Tachon et al., 2009). Its co-inoculation with probiotics in milk remarkably improved the viability of *Bifidobacterium* spp. (Yonezawa et al., 2010; Odamaki et al., 2011). Interestingly, *Lac. lactis* is a dairy starter culture used extensively in cheese fermentations and as a probiotic (Ruggirello et al., 2016; Jaskulski et al., 2020). The incorporation non-starter, adjunct lactic acid bacterial cultures in yogurt is a worthwhile proposition in the production of yogurt with enhanced health benefits (Ayivi and Ibrahim, 2022). However, the potential benefits must be balanced against any potential negative effects on yogurt fermentation kinetics, flavor and texture (Ayivi and Ibrahim, 2022). Generally, the combination of *Bifidobacterium* spp. with other probiotic lactic acid bacterial species have shown positive outcomes in terms of bioactive metabolites and therapeutic benefits (Pápai et al., 2021; Peng et al., 2022).

In addition, processes targeting the control of post-fermentation acidification in yogurt have been proposed for managing *Bifidobacterium* viability in yogurt. The H^+^-ATPase defective mutants of *L. delbrueckii* subsp. *bulgaricus* cannot exclude H^+^ protons and are sensitive to acidity due to their inability to maintain cytoplasmic pH homeostasis (Wang et al., 2013). As the primary organism responsible for post-fermentation acidification, the use of such mutants in yogurt fermentation can reduce the accumulation of acidity post-fermentation (Wang et al., 2013). Ongol et al. (2007) reported an enhanced viability of *B. breve* during storage in yogurt fermented with H^+^-ATPase defective mutants.

### 4.2 Stress adaptation

Stress adaptation involves the pre-exposure of an organism to sub-lethal stress conditions that induce the development of tolerance to subsequent lethal stress exposure (Fiocco et al., 2020). The process involves several repetitive generations of exposure to mild stress, punctuated by incremental stress intensification (Jiang et al., 2016). A typical stress adaptation process can involve up to 50 generations of repeated stress exposures in which stress-resistant variants are isolated every few generations (Jiang et al., 2016). Variants that exhibit a stable stress resistance phenotype are subsequently preserved as genetically adapted mutants (Berger et al., 2010). Some successful adaptation experiments have been reported for different *Bifidobacterium* species. An acid-resistant mutant strain of *B. longum* subsp. *longum* was isolated after a 50-generation successive subculturing in MRS broth adjusted to pH 2.5 (Jiang et al., 2016). Similarly, thermal stress- and oxidative stress-adapted bifidobacteria cells were isolated after successive exposures to heat and hydrogen peroxide, respectively (Berger et al., 2010; Mozzetti et al., 2010). The genetic basis of sustained stress adaptation is the evolutionary development of mutants that overexpress stress response genes. Using transcriptomic profiling of induced oxidative stress adaptation in *B. longum* subsp. *longum*, Xiao et al. (2011) observed an upregulation of genes encoding essential proteins involved in the protection or repair mechanisms of damaged cell components, such as alkyl hydroperoxide reductase C22 (AhpC), DNA-binding ferritin-like protein (Dps), ribonucleotide reductase (NrdA), and enolase. After successive heat shock treatments, Berger et al. (2010) also observed that heat-adapted mutants overexpressed the *dnaK* operon and the *clpB* gene in *B. longum*. Apart from the adaptive tolerance to the same stress factor used to induce adaptation (homologous adaptation), cross-protection to different stress factors (heterologous adaptation) can also occur (Chen et al., 2017). Central to the cross-tolerance is the role of the transcriptional general stress response regulators (Averina et al., 2012). In *Bifidobacterium* species, the WhiB-like proteins encoded by the *whiB* gene are the universal transcriptional regulators of stress response genes (Averina et al., 2012). As pleiotropic regulators, the effect of any induced stress adaptation can likely overlap among different stress factors (Averina et al., 2012).

### 4.3 Microencapsulation

Microencapsulation involves the use of biopolymers that entrap bacterial cells in a polymer matrix prepared into microgel spheres (Frakolaki et al., 2021). The entrapment within the microgel particles protects probiotics against environmental stress and aids their survival during food processing and storage (Yeung et al., 2016). While several biopolymers can be used in the preparation of probiotic microcapsules, alginate is by far the most widely used food-grade biopolymer (Liu et al., 2019; Abbas et al., 2022). A naturally occurring polysaccharide extracted from brown algae, alginate consists of a linear polymer of α-L-guluronic acid (G) and β-D-mannuronic acids (M) units in repetitive blocks (G-blocks and M-blocks) (Alba and Kontogiorgos, 2019). Solutions of alginates readily form gels in the presence of divalent cations such as Ca^2+^ through their interaction with the G-blocks in an egg-box model (Alba and Kontogiorgos, 2019). The microgel preparation process usually involves a solution of alginate mixed with a bacterial suspension that is subjected to extrusion to form droplets that are instantaneously hardened by treatment with CaCl_2_ solution into three-dimensional gel spheres entrapping the probiotic bacteria (Liu et al., 2019; Abbas et al., 2022). Another commonly used method in the preparation of alginate gel droplets is emulsion. This method involves the alginate-bacterial mixture suspended in an oil bath with a surfactant to produce a water-in-oil emulsion (Frakolaki et al., 2021). The emulsion is subsequently treated with a CaCl_2_ solution, and the formed beads are harvested by centrifugation (Liu et al., 2019). The gel spheres prepared by either of these methods are subsequently freeze-dried (Liu et al., 2019). Several studies have shown that encapsulation enhances the survival ability under environmental and simulated GIT stress conditions (Yeung et al., 2016; Ji et al., 2019; Cedran et al., 2021; Zhang et al., 2021). For instance, alginate- and chitosan-coated/alginate-encapsulated *B. longum* showed a 0.20–1.72 log_10_ CFU/g viability loss at 55–65°C compared to unencapsulated cells that had a viability loss of 3.0–5.0 log_10_ CFU/g loss (Ji et al., 2019). When subjected to simulated gastric conditions (pH 2.5), the viability of chitosan-coated/alginate-encapsulated *B. longum* decreased by 1.27 log_10_ CFU after 120 min compared to the viability of unencapsulated cells that had declined to undetected levels after the same time period (Ji et al., 2019). Besides the observed enhancement of survival under simulated stress conditions, the benefit of encapsulation has also been demonstrated in alginate-encapsulated *Bifidobacterium* spp. incorporated into yogurt and other foods. Cedran et al. (2021) observed a two-fold loss of viability of unencapsulated *B. lactis* compared to the alginate-encapsulated cells in jam after 6 days of storage. Similarly, Mousa et al. (2023) reported a higher viability of *B. bifidum* encapsulated in a double layer of whey protein and alginate in set yogurt during a 14-day storage at 4°C than free cells. Pradeep Prasanna and Charalampopoulos (2019) observed a > 3.0 log_10_ CFU/g viability decline for unencapsulated *B. animalis* subsp. *lactis* in goat milk yogurt over 28 days while the viability of alginate-encapsulated cells remained stable. However, it is worth noting that while the benefits of encapsulation indicate a better survival compared to unencapsulated cells, a general decline in viability especially over the duration of shelf life still occurs (Yeung et al., 2016; Mousa et al., 2023). Moreover, a disproportionately large number of encapsulation studies have been based on *B. animalis* subspp. *lactis*, an intrinsically acid and oxidative stress tolerant strain. Hence, there is still need for viability retention methods for the more stress-sensitive species like *B. bifidum*. Besides the viability retention benefits, the effects on microencapsulation on yogurt texture are as important. Mousa et al. (2023), observed an increase in viscosity, gumminess, chewiness, and adhesiveness in yogurt with alginate-encapsulated *B. bifidum*. Similarly, Li H. et al. (2021) observed an increase in water holding capacity and cohesiveness of yogurt with microcapsules of *L. paracasei*. Importantly most of the changes in texture are beneficial in yogurt quality and the microcapsule incorporated yogurt have been reported to be acceptable by sensory panels (Dimitrellou et al., 2019; Mousa et al., 2023).

### 4.4 Protective agents

Among the physicochemical stress factors associated with yogurt processing and storage, oxidative stress has the most significant effect on viability of *Bifidobacterium* spp. (Bolduc et al., 2006). Hence, the ability to control the dissolved oxygen content and the redox potential of yogurt can provide a sustainable approach to preserve viability. The incorporation of oxygen-scavenging compounds, such as ascorbic acid and cysteine, has been shown to improve *Bifidobacterium* spp. survival in yogurt (Norouzbeigi et al., 2021). At 500 mg/l, cysteine can maintain a negative redox potential in yogurt for 30 days (Dave and Shah, 1997a). However, despite its strong reducing capacity, cysteine is considered unsuitable for use in yogurt, as it impacts a sulfur taste (Meybodi et al., 2020). Regarding sensory effects, ascorbic acid is more compatible with dairy products (van Aardt et al., 2005). However, the antioxidant capacity of ascorbic acid is hindered by the gradual loss of its stability over the storage shelf life of yogurt (Dave and Shah, 1997b). From the highest concentration of 250 mg/Kg, only 15–20% of the ascorbic acid was retained in yogurt after 35 days of storage at 4°C (Dave and Shah, 1997b). An additional approach to protect *Bifidobacterium* spp. from the effects of oxidative stress is the addition of glucose oxidase and glucose in yogurt (Cruz et al., 2012a). Glucose oxidase utilizes oxygen as it oxidizes D-glucose to gluconic acid and hydrogen peroxide, thus causing a reduction in the dissolved oxygen content of yogurt (Afjeh et al., 2019). Cruz et al. (2012a) reported a significant increase in the viable population of *B. longum* in yogurts added with glucose oxidase and glucose. While the activity of glucose oxidase reduces the oxygen content of the yogurt, the release of hydrogen peroxide as a by-product has a negative impact on *Bifidobacterium* spp. Hence, the protective effect of the glucose oxidase + glucose system requires the addition of catalase as an accessory enzyme to eliminate the toxic effects of hydrogen peroxide (Cruz et al., 2012b). The use of protective agents in preserving probiotic viability is a technique that is already extensively used in spray-drying and freeze-drying processes for probiotic microencapsulation (Fiocco et al., 2020). A diverse range of substances have shown to be effective as protective agents against the osmotic, heat and cold stresses associated with spray- and freeze-drying processes (Fiocco et al., 2020). Some of these substances, such as skim milk powder, are milk by-products that are readily acceptable as ingredients in yogurt making. When used as a protectant, skim milk solids stabilize the bacterial cell membrane by forming a protective coating on cell wall proteins (Terpou et al., 2019). This prevents cell damage due to thermal and osmotic stress associated with spray drying (Terpou et al., 2019). Other substances, such as sugars, sugar alcohols and complex carbohydrates, have proven to be effective cryoprotectants during freeze-drying (Rockinger et al., 2021). Although they are used as protective agents against freezing, during freeze drying, cryoprotective agents can be valuable in preserving *Bifidobacterium* viability during the long period of cold stress during yogurt shelf-life. Among the cryoprotective agents, trehalose and glycerol are the most favorable for use in yogurt. These compounds lower the phase transition temperature of the cell membrane under cold conditions, thus maintaining it in a flexible liquid crystalline state while also keeping the cell membrane hydrated through their hydrogen bond interactions with phospholipid heads (Rockinger et al., 2021). Furthermore, due to their water-binding abilities, the compounds can suppress ice nucleation and prevent the damaging effect of ice crystal formation (Rockinger et al., 2021).

## 5 *Bifidobacterium* viability determination methods

### 5.1 Culture-based methods

In the last 20 years, a broad range of culture media have been proposed for the enumeration of *Bifidobacterium* spp. in dairy products (Van de Casteele et al., 2006; Lima et al., 2009; Ashraf and Smith, 2015). Among them, is MRS-NNLP agar, a widely used selective media (Dave and Shah, 1996; Ashraf and Shah, 2011; Karimi et al., 2012). In such media, the use of selective supplements may lead to an underestimation of viability as some live *Bifidobacterium* spp. cells may be sensitive to the selective agents (Dave and Shah, 1997a). For example, Van de Casteele et al. (2006), reported a lower recovery of *Bifidobacterium* spp. on MRS-NNLP agar than on MRS agar. Secondly, the selectivity of the medium depends on the type of non-target species present in the product (Van de Casteele et al., 2006; Ashraf and Smith, 2015). In mixed species products with *L. rhamnosus* and *L. acidophilus* strains, MRS-NNLP agar could not select for *Bifidobacterium* spp. (Van de Casteele et al., 2006; Ashraf and Smith, 2015). Currently, the International Organization of Standardization (ISO) and International Diary Federation (IDF) recommended culture-based method for the enumeration of *Bifidobacterium* spp. in dairy products (ISO 29981:2010/IDF 220:2010) is based on *trans-*galactosylated oligosaccharides (TOS) propionate agar containing lithium mupirocin as a selective agent (TOS-Mup media) (International Organization for Standardization [ISO], and International Dairy Federation [IDF], 2010). Like MRS-NNLP agar, TOS-Mup media has a low recovery for some *Bifidobacterium* spp. (Bunesova et al., 2015). Table 2 summarizes the recent applications of the recommended media for selective enumeration of *Bifidobacterium* spp. in yogurt and other dairy-based products. It is evident that despite all the intensive work, there is still a need for a medium that could be used as a standard for the quantification of all *Bifidobacterium* spp. Moreover, species and strain-specific physiological requirements affect quantification efficiency (Van de Casteele et al., 2006; Bunesova et al., 2015). In addition to the drawbacks mentioned above, culture-based methods are laborious and have long results turnaround time of up to 72 h as agar plates need to be incubated under specific growth conditions (Davis, 2014; Geng et al., 2014). Since these methods are based on the cultivability of the cells, they cannot quantify cells that are in a viable but non-culturable (VBNC) state (Jackson et al., 2019; Vinderola et al., 2019). Hence, culture-based methods may underestimate viable counts of beneficial probiotic bacteria (Davis, 2014).

**TABLE 2:** Summary of the recent applications of the recommended media used for the selective enumeration of Bifidobacterium spp. in yogurt, lyophilized cultures, and other dairy-based products.

Base	Selective supplement	Species mixture	Target *Bifidobacterium* spp.	Product	References
MRS	NNLP (Nalidixic acid, neomycin sulfate, Lithium chloride and paromomycin sulfate)	*L. acidophilus, B. animalis subsp. lactis* BB-12, and *S. thermophilus*	*B. animalis* subsp. *lactis* BB-12	Fruited yogurt	Erkaya-Kotan, 2020; Najgebauer-Lejko et al., 2021
MRS	NNLP (Nalidixic acid, neomycin sulfate, Lithium chloride and paromomycin sulfate), 0.3% v/v L-cysteine HCl	*L. delbrueckii subsp. bulgaricus, B. animalis subsp. lactis* BB-12, and *S. thermophilus*	*B. animalis* subsp. *lactis* BB-12	Yogurt	Akalin A. S. et al., 2018; Frakolaki et al., 2022
MRS	NNLP (Nalidixic acid, neomycin sulfate, Lithium chloride and paromomycin sulfate), and L-cysteine	*Debaryomyces hansenii, Lactococcus cremoris, L. lactis, L. diacetylactis, Leuconostoc spp., S. thermophilus* and *B. bifidum* BB-11	*B. bifidum* B-11	Kefir	Buran et al., 2021
MRS	5% *v/v* NNLP (15 mg Nalidixic acid, 100 mg neomycin sulfate, 3 g Lithium chloride and 200 mg paromomycin sulfate) and 3% *v/v* L-cysteine HCl	*L. acidophilus* La-5*, B. animalis subsp. lactis* BB-12*, S. thermophilus and L. delbrueckii subsp. bulgaricus*	*B. animalis* subsp. *lactis* BB-12	Plain and Flavored Yogurts	Tsevdou et al., 2020
MRS	NNLP (Nalidixic acid, neomycin sulfate, Lithium chloride and paromomycin sulfate), and L-cysteine HCl	*B. bifidum* PTCC 1644 and ATCC 29521*, L. delbrueckii subsp. bulgaricus* and *S. thermophilus* ST-20Y	*B. bifidum* PTCC 1644 and ATCC 29521	Yogurt	Ghaderi-Ghahfarokhi et al., 2021
TOS Propionate	MUP (Mupirocin)	*B. animalis subsp. lactis* BB-12 and *Propionibacterium shermanii subsp. freudenreichii*	*B. animalis* subsp. *lactis* BB-12	Dairy Drink	Yerlikaya et al., 2020
MRS	LP (0.3% Lithium chloride, 0.05% L-cysteine HCl and 0.9% sodium propionate)	*L. acidophilus, B. longum*, and *S. thermophilus*	*B. longum*	Yogurt	Zhang et al., 2019
MRS	MUP (Lithium Mupirocin)	YF-L812 starter cultures *(L. delbrueckii subsp. bulgaricus* and *S. thermophilus) and B. animalis subsp. lactis* BB-12	*B. animalis* subsp. *lactis* BB-12	Yogurt	He J. et al., 2019
Bifidobacteria Selective Media (BSM)		*L. acidophilus* DSMZ 20079*, B. bifidum* DSMZ 20456*, L. bulgaricus*, and *S. thermophilus*	*B. bifidum* DSMZ 20456	Flavored Yogurt	Turgut and Cakmakci, 2018
MRS	NNLP (Nalidixic acid, neomycin sulfate, Lithium chloride and paromomycin sulfate)	*L. acidophilus* and *B. animalis subsp. lactis*	*B. animalis* subsp. *lactis* BB-12	Ice Cream	Akalin A. et al., 2018
MRS	0.05 mg/mL MUP and, 0.05% L-Cysteine	*L. paracasei* PC-01 and *B. animalis subsp. lactis* Probio-M8	*B. animalis* subsp. *lactis* Probio-M8	Fermented Milk Beverage	Hao et al., 2023

### 5.2 Flow cytometry

Flow cytometry is a single-cell analysis technique that is used to explore the physical and physiological characteristics of microbial cells as they pass through a beam of light (usually blue laser, 488 nm) (Davis, 2014). When used with fluorescent staining, the technique can distinguish between live and dead cells based on viability markers such as membrane integrity and intracellular enzyme activity (Wendel, 2022). Cell integrity is often determined by double staining with the DNA binding dyes such as diamidinophenylindole (DAPI), acridine orange and the SYTO dye series, which emits green fluorescence after excitation with 488 nm laser and propidium iodide (PI), which emits red fluorescence after excitation at the same wavelength (Veal et al., 2000). The exclusion of PI by cells with intact membranes gives viable cells a green fluorescence, while non-viable cells with damaged membranes fluoresce red (Veal et al., 2000). Intracellular enzyme activity is often determined using membrane-permeant fluorogenic substrates such as 5,6-carboxyfluorescein diacetate (5,6-cFDA), which, upon enzymatic hydrolysis by intracellular esterases from live cells, release a green, fluorescent carboxyfluorescein (Hoefel et al., 2003). Following a gating for live- (green fluorescent) and dead-cell (red fluorescent) subpopulations, viability determination is then based on the enumeration of cells from an appropriately diluted sample that falls within the live-cell region (Foglia et al., 2020). Although FCM has been available for a long time as a high throughput method of studying bacterial cell viability, its use in the enumeration of probiotic viability in foods has been limited. So far, one protocol by the ISO and IDF (ISO 19344 – IDF 232: 2015) is available for the flow cytometric enumeration of starter cultures and probiotics in fermented products (International Organization for Standardization [ISO], and International Dairy Federation [IDF], 2015). However, the non-specificity of the method due to its inability to selectively enumerate viable probiotics in the presence of the starter cultures, is a significant limitation. Some studies have attempted to improve the species selectivity of flow cytometry by incorporating antibody labeling in conjunction with membrane integrity and enzyme activity fluorescent probes (Geng et al., 2014; Chiron et al., 2018). The immuno-flow cytometry assay utilizes the specific binding of a primary polyclonal antibody to ligands on the bacterial cell, which is subsequently bound to a secondary antibody conjugated to a fluorescent tag and further stained with a viability probe (Wilkinson, 2018). Using this concept of dual labeling with polyclonal antibodies and 5,6-cFDA, only viable *B. lactis* were enumerated from mixed cultures and fermented products containing *L. bulgaricus*, *S. thermophilus* and *Lac. lactis* (Geng et al., 2014). Similarly, polyclonal antibodies specific for *B. bifidum*, *B. longum* subsp. *infantis*, *B. longum* subsp. *longum*, *L. helveticus* and *L. rhamnosus* in combination with SYTO^®^24 and PI staining were used to selectively enumerate the viable individual strains in multi-strain probiotic products (Chiron et al., 2018). Another immuno-flow cytometry concept with a potential application in probiotic enumeration is the immunomagnetic separation of specific probiotic strains from a mixed species using antibody-coated magnetic beads (Wilkinson, 2018). This technique, commonly used for the recovery and enrichment of pathogens, was recently used to isolate *L. paracasei* from human feces (Takada et al., 2023). When applied for probiotic viability enumeration, recovered cells from immunomagnetic separation can be analyzed by flow cytometry after staining with viability probes (Wilkinson, 2018). Apart from its value in viability determination, flow cytometry offers other benefits in studying some of the physical and physiological characteristics of viable cells that could relate to their stress responses and probiotic functionalities (Wendel, 2022). The additional element of flow cytometry, fluorescence-activated cell sorting (FACS), allows for the isolation and recovery of different subpopulations from a flow cytometry assay (viable, dead, and injured cells) for further analysis of metabolic, physiological, and genetic characteristics relating to probiotic functionality (Wendel, 2022).

### 5.3 Molecular and next generation methods

#### 5.3.1 qPCR

Real-time quantitative polymerase chain reaction (qPCR)-based methods are premised on the detection and amplification of DNA of target organisms using fluorescent DNA intercalating dyes (e.g., SYBR green) or sequence-specific fluorogenic probes (e.g., TaqMan probes) (Agrimonti et al., 2019; Ruijter et al., 2021). Sequence-specific oligonucleotide primers are used to flank specific fragments of a target gene to be amplified. As the amount of PCR amplicon increases during PCR, the fluorescent signal accumulates. The quantification cycle (Cq) is then measured in the exponential phase of qPCR when the fluorescence signal has accumulated above the background fluorescence (Davis, 2014). During exponential phase, the amount of PCR amplicon is directly proportional to the DNA template (Davis, 2014). Hence, using standard curves established by plotting the Cq values against DNA copies, the number of copies of target species in the food sample can be determined (Davis, 2014). While the 16S rRNA gene has frequently been used in experimental qPCR-based methods (Zhang and Fang, 2006; Kim H. B. et al., 2020), its use poses challenges for quantification as it may exist as more than one copy in some bacterial genomes and it has a high sequence similarity between the *Bifidobacterium* spp. (Kim H. B. et al., 2020; Fan et al., 2021; Shi et al., 2022). For reliable quantification, the target gene should be a single copy within the bacterial genome (Shi et al., 2022). Recently, protein-encoding housekeeping genes such as the translation elongation factor EF-TU (*tuf*) and phenylalanine tRNA ligase subunit alpha (*pheS*) genes have been successfully used in qPCR methods for probiotic quantification in dairy products (Scariot et al., 2018; Fan et al., 2021; Shi et al., 2022). Alternatively, comparative genomics can be used to find unique and specific genetic markers for primer design and selective detection of closely related species and subspecies, especially of *Bifidobacterium*, where the use of housekeeping genes may be limited (Kim H. B. et al., 2020). A recent study by Kim H. B. et al. (2020) successfully designed species and subspecies-specific primers for 22 *Bifidobacterium* species and subspecies based on the genetic markers identified using comparative genomics. The main challenge of qPCR-based methods, however, is their inability to differentiate between DNA from live and dead cells (Scariot et al., 2018; Shehata and Newmaster, 2021; Guo et al., 2022). This implies that the use of qPCR methods may overestimate counts. Therefore, its application in probiotic viability determination is limited unless coupled with another technique that allows selective quantification of viable counts.

#### 5.3.2 Propidium monoazide qPCR for quantification of *Bifidobacterium* spp. in yogurt

The challenge of distinguishing between live and dead cells encountered with general qPCR methods can be circumvented with the inclusion of viability dyes (Scariot et al., 2018; Shi et al., 2022; Shehata et al., 2023). At present, there are three types of viability dyes used for the selective quantification of viable bacterial cells, namely ethidium monoazide (EMA), propidium monoazide (PMA), and PMAxx, an improved version of PMA (Lv et al., 2021; Shehata and Newmaster, 2021; Mu et al., 2022). PMA is a next-generation viability dye developed in 2006 to overcome the challenges of EMA, which was found to penetrate cell membranes of live cells of some bacterial species (Nocker et al., 2006).

##### 5.3.2.1 PMA mechanism of action

Propidium monoazide was produced through the chemical modification of propidium iodide by replacing the amino group on the phenanthridine ring with the azide group that can form a covalent crosslink with the DNA (Nocker et al., 2006; Cangelosi and Meschke, 2014). Recently, an improved and more effective version of PMA with the same spectral properties, PMAxx, was developed by Biotium Inc. PMAxx is a new generation DNA intercalating and membrane impermeant dye that can only penetrate the cell membranes of dead cells (Guo et al., 2022; Kallastu et al., 2023). The procedure for the PMAxx-based method involves an initial stage of incubation of the food sample with about 25–150 μM of PMAxx in the dark to allow the dye to penetrate compromised cell membranes and intercalate with the DNA (Nocker et al., 2006; Mu et al., 2022). Upon exposure to bright light, the azide group of PMAxx produces nitrene, a highly photo-reactive molecule, that forms a covalent crosslink with DNA or reacts with water to form hydroxylamine (an inactivated form of PMAxx) (Nocker et al., 2006; Fittipaldi et al., 2012). The PMAxx-DNA conjugate is insoluble and, therefore, is removed with cell debris during DNA extraction, while the remaining conjugates are not amplified during PCR (Nocker et al., 2006). Hence, only DNA from live cells is amplified during qPCR.

##### 5.3.2.2 Application of PMA-qPCR method for quantification of *Bifidobacterium* spp. in yogurt

A few studies have reported the application of PMA-qPCR methods for quantification of *Bifidobacterium* spp. in fermented dairy products (Table 3). PMA-qPCR methods can provide insight into the physiological state of probiotics due to their ability to detect cells in the VBNC state (Dong et al., 2020). Kibbee and Örmeci (2017) reported the application of PMA-qPCR for quantification of VBNC cells of *E. coli* in wastewater effluents, while Guo et al. (2021) used the same method for VBNC cell enumeration in drinking water. *Bifidobacterium* spp. can exhibit the VBNC state as a protective mechanism when under stressful environments (Lahtinen et al., 2008). For example, *B. longum* and *B. animalis* subsp. *lactis* were found to exhibit the VBNC state under acidic conditions in fermented products (Lahtinen et al., 2008). The presence of VBNC *Bifidobacterium* spp. in fermented dairy products, can lead to an underestimation of viability, a key parameter in probiotic quality assurance. Several factors must be considered for the effective application of PMA-qPCR methods in yogurt. A review by Fittipaldi et al. (2012) identified factors that can affect the efficiency of PMA-qPCR methods. These factors include probiotic species type, dye concentration, pH and turbidity of the sample. The authors recommended a pH adjustment and dilution for highly acidic (pH ≤ 4) and turbid (≥10 Nephelometric Turbidity Units, NTU) samples, respectively. Several PMA-qPCR studies have included the pretreatment step of fermented dairy products before PMA treatment to disperse the casein micelles and to adjust the pH to 6.5 (Scariot et al., 2018; Yang et al., 2021). In addition, PMA treatment in these studies was carried out in clear media such as water or phosphate-buffered saline (PBS). However, the inclusion of this pretreatment step is not consistent with the PMA-qPCR methods for the quantification of probiotics in dairy-fermented products. For example, Shi et al. (2022) added PMA directly to the fermented milk sample (1 mL) without including a pretreatment step. In all these studies, PMA-qPCR methods selectively quantified live probiotic cells in fermented dairy products (Scariot et al., 2018; Yang et al., 2021; Shi et al., 2022). This shows that PMA may be applied directly to fermented dairy milk. However, additional PMA-qPCR studies on fermented dairy milk are necessary to support this conclusion and to comprehend the impact of fermented milk product pH, such as yogurt, on PMA efficacy.

**TABLE 3:** Summary of culture-independent techniques that have been used for the determination of Bifidobacterium spp. viability in yogurt and other dairy-based products.

Dairy-based probiotic food samples					
**Method**	**Product**	**Species mixture**	**Target species**	**Findings**	**References**
PMA-qPCR	Fermented milk	*S. thermophilus, L. delbrueckii subsp. bulgaricus, L. casei, L. acidophilus and B. lactis*	*B. lactis* (BB-12)	Viable counts comparable to plate count (Pearson correlation coefficient = 0.995). Rapid (results obtained within 3 h).	García-Cayuela et al., 2009
EMA-qPCR	Yogurt	*S. thermophilus, L. delbrueckii subsp. bulgaricus and B. longum* ATCC 15707	*B. longum* ATCC 15707	Viable counts slightly lower than plate counts. Good correlation between the two methods (*R*^2^ = 0.9948). Rapid (results obtained within 4 h).	Meng et al., 2010
PMA-qPCR	Cheddar cheese	*Lactococcus* spp., *L. rhamnosus* RO011, *L. helveticus* RO052, and *B. animalis* subsp. *lactis* BB-12	*B. animalis* subsp. *lactis* BB-12	Viable counts higher than plate counts during cheese manufacturing.	Desfossés-Foucault et al., 2012
Flow Cytometry (FCM)	Fermented milk	*L. lactis* CNCM I-1631., *L. delbrueckii* subsp. *bulgaricus* CNCM I-1519, *S. thermophilus* CNCM I-1630, and *B. animalis* subsp. *lactis*	*B. animalis* subsp. *lactis*	Viable counts comparable to plate counts (correction coefficient = 0.954). Uses species-specific polyclonal antibody. Rapid (results obtained within 2 h).	Geng et al., 2014
PMA-qPCR	Synbiotic ice-cream	*L. acidophilus* LA-5, and *B. animalis* subsp. *lactis* BB-12	*B. animalis* subsp. *lactis* BB-12	Application of PMA-qPCR. Reliable method for quantification of probiotics under stressful environments.	Matias et al., 2016
PMA-qPCR	Petit-suisse cheese	*L. acidophilus* LA-5, *B. animalis* subsp. *lactis* BB-12 and *S. thermophilus*	*B. animalis* subsp. *lactis* BB-12	Application of PMA-qPCR.	Padilha et al., 2016
PMA-qPCR	Synbiotic table spread	*Bifidobacterium* BB-12	*Bifidobacterium* BB-12	Good correlation between PMA-qPCR with plate counts (*r* = 0.92 to 0.97). Results of the two methods generally comparable	Dos Santos et al., 2018
**Lyophilized Culture Samples**					
Chip-Based dPCR coupled with PMA	Lyophilized cultures	*L. acidophilus* NCFM and *B. animalis* subsp. *lactis* BI-04	*B. animalis* subsp. *lactis* BI-04	Counts slightly lower but comparable to plate counts. Low variation between results compared to plate count method. Rapid (results obtained within 1 h) and enumeration at strain level.	Hansen et al., 2018
PMA-qPCR	Lyophilized capsules	*L. acidophilus* LA-5 and *B. animalis* subsp. *lactis* BB-12	*B. animalis* subsp. *lactis* BB-12	Viable counts comparable to plate count.	Kramer et al., 2009
Flow Cytometry (FCM)	Freeze-dried probiotic cultures	*L. rhamnosus* R0011, *L. helveticus* R0052, B. *longum* subsp. *longum* R0175 and *Saccharomyces cerevisiae* var *boulardii* CNCM I-1079	*B. longum* subsp. *longum* R0175	Counts generally higher than plate counts (in 73% cases). Good correlation (R^2^) of 0.8222 between the two methods. Uses species-specific polyclonal antibody. Rapid (results obtained within 2 h)	Chiron et al., 2018
		*L. helveticus* R0052, *B. longum* subsp. *infantis* R0033 and B. *bifidum* R0071	*B. longum* subsp. *infantis* R0033 and *B. bifidum* R0071		
Droplet dPCR coupled with PEMAX	Freeze-dried probiotic powders	*B. animalis* subsp. *lactis* BI-04	*B. animalis* subsp. *lactis* BI-04	Relative difference of 15% between the ddPCR and plate count. Good correlation (r) of 0.76 between the two methods. Low variation between results compared to plate count method	Hansen et al., 2020
		*B. animalis* subsp. *lactis* HN019	*B. animalis* subsp. *lactis* HN019		
		*B. animalis* subsp. *lactis* Bi-07	*B. animalis* subsp. *lactis* Bi-07		
Droplet dPCR coupled with PE51	Freeze-dried probiotic cultures	*B. animalis* subsp. *lactis* Bi-07	*B. animalis* subsp. *lactis* Bi-07	Used PE51 dye made from a combination of EMA and PMA. Good correlation between PE51-ddPCR with plate counts (*r* = 0.762), and better than PMA-ddPCR and EMA-ddPCR	Kiefer et al., 2022
		*B. animalis* subsp. *lactis* BI-04	*B. animalis* subsp. *lactis* BI-04		

#### 5.3.3 Digital PCR

Digital PCR (dPCR) is a third-generation PCR and an emerging technology for microbial quantification (Agrimonti et al., 2019; Kiefer et al., 2022). Unlike qPCR, dPCR does not require standard curves for absolute quantification, and its sample preparation and amplification confirmation methods are different (Salipante and Jerome, 2020). In dPCR, a PCR reaction mixture containing the target sequence is partitioned randomly into thousands of small individual microreactors, such as oil droplets and chip wells (Hansen et al., 2020; Salipante and Jerome, 2020; Lv et al., 2021). A signal fluorescence from partitions containing a single copy of the target sequence is measured at the end of a PCR run (Hansen et al., 2018, 2020; Salipante and Jerome, 2020). Poisson statistics based on comparing the number of positive (with signal fluorescence) and negative reactions is used to determine the absolute quantity of the target sequence (Hansen et al., 2020; Salipante and Jerome, 2020). Like normal qPCR, dPCR cannot distinguish between live and dead cells; hence, it is coupled with viability dyes for selective quantification of live cells (Kiefer et al., 2022). Studies have reported the application of dPCR methods for quantification of *Bifidobacterium* spp. and other lactic acid bacterial species, mainly in freeze-dried products, indicating a good correlation with plate counts (Summarized in Table 3). In addition, dPCR methods hold several advantages over culture-based methods. These include short results turnaround time, low variation between results and the ability to quantify probiotics at strain levels (Hansen et al., 2020). However, high DNA concentrations can affect probiotic quantification by dPCR (Kim et al., 2023). For example, a recent study quantifying probiotic *L. casei* in milk showed that quantification at high DNA concentrations was not possible as dPCR was saturated and resulted in a narrow linear dynamic range (Kim et al., 2023). However, this can be solved by diluting the sample (Kim et al., 2023).

#### 5.3.4 Next generation sequencing methods

Although the application of next generation sequencing (NGS) methods in the study of food microbiomes has been widely reported (Cao et al., 2017; Jagadeesan et al., 2019), their use as quality assurance tools for probiotic viability in foods is still limited. NGS applications in foods have mainly been limited to metagenomic analysis that provides data on relative abundance of different taxa (Cao et al., 2017). Despite the high-resolution ability of NGS methods, the inability to provide information on the absolute quantities and viability status of the organisms in the food has limited its use in viability enumeration. Interestingly, some recent studies have shown the potential application of NGS in viability determination when the technology is coupled with a viability dye (Kallastu et al., 2023). Using the 16S rRNA gene amplicon sequencing coupled with PMAxx reagent, Kallastu et al. (2023) elaborated a workflow for the determination of absolute numbers of viable organisms in *kimchi* and sauerkraut. The developed workflow involved the addition of a spike-in control (standard) into the sample following PMAxx treatment. After the 16S rRNA gene amplicon sequencing, the number of viable bacteria was determined from the relative abundance and the ratios of the spike-in reads (Kallastu et al., 2023).

### 5.4 Single-cell Raman spectroscopy (SCRS)

Culture-independent, label-free, non-invasive, rapid single-cell Raman spectroscopy (SCRS)-based techniques that give a collective insight into the organism’s phenome and genome are emerging as potential methods of microbial characterization (Jayan et al., 2022; Zhang et al., 2023). Recently, a novel automated SCRS-based technique that combines single-cell identification, viability, vitality and sequencing (SCIVVS) for the characterization of probiotics including *Bifidobacterium* spp., was described by Zhang et al. (2023). In the SCIVVS technique, probiotic characterization is a stepwise process where cells are first harvested from the probiotic sample, treated with a stable isotope probing (SIP), namely, deuterium oxide (D_2_O), which can only be taken up by live cells and subjected to Raman spectroscopy at single-cell resolution (Zhang et al., 2023). Other isotopes, such as ^13^C and ^15^N, respectively, can also be used for SIP in SCRS-based techniques (Jayan et al., 2022). SIP is based on the principle that the Raman spectra shift when an atom is substituted with its heavier isotope (Jayan et al., 2022). Hence, in the case of H_2_O and D_2_O (heavy water), the uptake of H_2_O in live and metabolically active cells is in the silent region (2040–2300 cm^–1^) of SCRS (He Y. et al., 2019). The uptake of D_2_O by the cells results in the partial replacement of the hydrogen (H) atom with the deuterium atom (D) (He Y. et al., 2019; Jayan et al., 2022). This results in the production of C – D bands, which can be modeled to deduce the metabolic state and viability of the cell (He Y. et al., 2019; Zhang et al., 2023). Hence, the SCRS of D_2_O-treated cells can be used to determine probiotic viability, vitality, and species-level identification (based on a compiled SCRS database of reference species) (Zhang et al., 2023). SCIVVS is also coupled with a single-cell assembly genome sequencing and thus gives comprehensive analysis from genome to phenome (Zhang et al., 2023). However, more studies are needed on applying and assessing the suitability of SCRS-based techniques for probiotic quantification.

## 6 Conclusion and future perspectives

*Bifidobacterium* species are one of the most important members of the GIT of healthy humans, with a substantial body of evidence showing their beneficial probiotic functionalities in experimental models and human trials. Notably, while evidence has shown that some physiological effects of probiotics, such as immunomodulatory properties, can be elicited by bacterial cell components like lipoteichoic acids and peptidoglycan, which can be constituents of both live and dead cells, some probiotic functionalities are dependent on viability and are species and strain specific. Therefore, as envisioned in the definition, viability is a critical quality assurance parameter for probiotics and probiotic foods. Unlike other common probiotic organisms such as lactic acid bacteria, the incorporation and sustained survival of bifidobacteria in probiotic carrier foods like yogurt is a significant challenge. Yogurt process stress factors such as acidity, oxygen, heat, osmotically active solutes, and cold storage impede *Bifidobacterium* spp. survival and result in viability decline over the product shelf life. Although several studies have investigated the phenotypic responses of different *Bifidobacterium* spp. to these stress factors, molecular stress response mechanisms still need to be fully elucidated. Response mechanisms to osmotic and cold stress particularly, are yet to be deciphered. Understanding the bifidobacterial stress response mechanisms is critical to the development of strategies to preserve cell viability. Hence, approaches such as stress adaptation, process modifications, microencapsulation and the use of stress protective agents have been investigated, with varying levels of success, as viability retention methods. In addition to viability retention approaches, viability measurement is the ultimate quality assurance requirement for probiotic foods. However, the available standard culture-based methods have proved inadequate for accurately determining *Bifidobacterium* viability in probiotic yogurt.

To a limited extent, flow cytometry has been considered an alternative method for the viability determination of yogurt cultures. However, the method lacks specificity and has limited application for *Bifidobacterium* viability. New innovations in immuno- flow cytometry, where fluorescent viability staining is linked to monoclonal antibodies, are expected to improve the applicability of the technology. Moving forward, molecular-based methods such as PMA-qPCR, digital PCR and sequencing, represent the future generation of methods for viability determination. Already, metagenomic sequencing, is used in microbiome analysis and relative quantification in foods. Innovative approaches to adapt the next generation sequencing for absolute microbial and viability quantification by coupling the sequencing with viability dyes and spike-in controls represents novel methods for the future. The adaptation of a recently described novel next-generation method called single-cell identification, viability and vitality tests, and source-tracking (SCIVVS) utilizes a D_2_O-probed single-cell raman spectrum (SCRS) that can accurately quantify cell viability at the species level based on the C–D band. Crucially, these alternative methods with the accompanying techno-economic assessments, should be developed into standardized and validated protocols for industrial probiotic viability quality assurance applications.

## Author contributions

TS: Conceptualization, Formal analysis, Investigation, Methodology, Writing—original draft, Writing—review and editing. TM: Conceptualization, Formal analysis, Investigation, Methodology, Writing—original draft, Writing—review and editing. UT: Conceptualization, Formal analysis, Investigation, Methodology, Writing—original draft, Writing—review and editing. MT: Conceptualization, Funding acquisition, Project administration, Supervision, Writing—review and editing. EB: Conceptualization, Funding acquisition, Project administration, Supervision, Writing—review and editing.
